# Supplementary material for: Bridging prediction and reality: Comprehensive analysis of experimental and AlphaFold 2 full-length nuclear receptor structures
Source: Comput Struct Biotechnol J. 2025 May 15;27:1998–2013. doi: 10.1016/j.csbj.2025.05.010 (PMC12149446; doi:10.1016/j.csbj.2025.05.010)
Supplement: MMC 1 — Data for RMDS, SSE and Domain architecture analysis. [file mmc1.docx]

**Table 1. Comparison of human GR vs experimental (PDB: 7PRV, 7PRW) and predicted structures (AlphaFold2)**

|  | Experimental (GR-7PRV) | | Predicted (GCR -AlphaFold2) |
| --- | --- | --- | --- |
|  | Chain A | Chain B |  |
| super exp, predicted  (all-atom, CA, backbone)  DBD  LBD | 0.496 (455 to 455)  0.425 (69 to 69)  0.451 (268 to 268)  0.438 (1368 to 1368)  0.383 (198 to 198)  0.377 (770 to 770) | 0.484 (447 to 447)  0.404 (67 to 67)  0.445 (265 to 265)  0.539 (1373 to 1373)  0.449 (187 to 187)  0.453 (749 to 749) |  |
| get_distance (comDBD-comLBD) | 32.078 Angstroms. | 46.865 Angstroms. | 42.664 Angstroms |
| get_angle (com)  comDBD, comHinge, comLBD | 100.707 | 104.006 | 105.517 |
| get_dihedral  comDBD, last_DBD_atom, first_LBD_atom, comLBD | -88.633 | -12.502 | -73.475 |
| DBD ss%  LBD ss% | Helix content: 38.03%  Sheet content: 8.45%  Loop content: 53.52%  Helix content: 77.06%  Sheet content: 4.33%  Loop content: 18.61% | Helix content: 38.03%  Sheet content: 8.45%  Loop content: 53.52%  Helix content: 75.32%  Sheet content: 3.03%  Loop content: 21.65% | Helix content: 42.25%  Sheet content: 9.86%  Loop content: 47.89%  Helix content: 69.70%  Sheet content: 4.33%  Loop content: 25.97% |

|  | Experimental (GCR-7PRW) | | Predicted (GCR -AlphaFold2) |
| --- | --- | --- | --- |
|  | Chain A | Chain B |  |
| super exp, predicted  (all-atom, CA, backbone)  DBD  LBD | 0.481 (420 to 420)  0.412 (65 to 65)  0.417 (246 to 246)  0.559 (1487 to 1487)  0.507 (214 to 214)  0.508 (848 to 848) | 0.520 (437 to 437)  0.426 (65 to 65)  0.444 (250 to 250)  0.555 (1465 to 1465)  0.496 (206 to 206)  0.512 (828 to 828) |  |
| get_distance (comDBD-comLBD) | 32.330 Angstroms. | 44.134 Angstroms. | 42.664 Angstroms. |
| get_angle (com)  comDBD, comHinge, comLBD | 103.528 | 97.590 | 105.517 |
| get_dihedral  comDBD, last_DBD_atom, first_LBD_atom, comLBD | -91.144 | -6.825 | -73.475 |
| DBD ss%  LBD ss% | Helix content: 39.44%  Sheet content: 8.45%  Loop content: 52.11%  Helix content: 74.46%  Sheet content: 3.90%  Loop content: 21.65% | Helix content: 38.89%  Sheet content: 8.33%  Loop content: 52.78%  Helix content: 73.16%  Sheet content: 4.33%  Loop content: 22.51% | Helix content: 42.25%  Sheet content: 9.86%  Loop content: 47.89%  Helix content: 69.70%  Sheet content: 4.33%  Loop content: 25.97% |

**Table 2A. Comparison of human HNF4A experimental (PDB: 4IQR (A, E)) and predicted structures**

|  | Experimental (HNF4A-4IQR) | | Predicted (HNF4A -AlphaFold2) |
| --- | --- | --- | --- |
|  | Chain A | Chain E |  |
| super exp, predicted  (all-atom, CA, backbone)  DBD  LBD | 0.501 (464 to 464)  0.423 (70 to 70)  0.443 (270 to 270  0.422 (1392 to 1392)  0.370 (197 to 197  0.382 (783 to 783 | 0.368 (434 to 434)  0.282 (66 to 66)  0.293 (247 to 247)  0.468 (1426 to 1426)  0.397 (201 to 201)  0.406 (787 to 787) |  |
| get_distance (comDBD-comLBD) | 38.343 Angstroms | 38.661 Angstroms. | 65.058 Angstroms |
| get_angle  comDBD, comHinge, comLBD | 108.252 | 119.897 | 170.601 |
| get_dihedral  comDBD, last_DBD_atom, first_LBD_atom, comLBD | -99.935 | -112.931 | -166.304 |
| ss%  DBD  LBD | Helix  : 34.67%  Sheet  :  9.33%  Loop  :  0.00%  Helix  : 68.58%  Sheet  :  3.98%  Loop  :  0.00% | Helix  : 36.00%  Sheet  :  9.33%  Loop  :  0.00%  Helix  : 69.03%  Sheet  :  3.98%  Loop  :  0.00% | Helix  : 40.00%  Sheet  :  9.33%  Loop  :  0.00%  Helix  : 70.80%  Sheet  :  3.98%  Loop  :  0.00% |

RMSD values are low for superimposed 4NQA (A, E chains) and predicted structure, but there is a quite big difference in DBD-LBD distance, and therefore angles. Predicted structure has slightly higher helix content in DBD (40% vs 35-36%).

**Table 2B. Comparison of human HNF4A experimental (PDB: 4IQR (B, F)) and predicted structures**

|  | Experimental (HNF4A-4IQR) | | Predicted (HNF4A -AlphaFold2) |
| --- | --- | --- | --- |
|  | Chain B | Chain F |  |
| super exp, predicted  (all-atom, CA, backbone)  DBD  LBD | 0.369 (442 to 442)  0.305 (70 to 70)  0.320 (265 to 265  0.379 (1408 to 1408)  0.324 (195 to 195)  0.335 (761 to 761) | 0.360 (429 to 429)  0.288 (67 to 67)  0.300 (251 to 251)  0.452 (1332 to 1332)  0.403 (190 to 190)  0.417 (749 to 749) |  |
| get_distance (comDBD-comLBD) | 66.265 Angstroms | 66.377 Angstroms | 65.058 Angstroms |
| get_angle  DBD, mid, LBD  DBD, LBD, mid  Mid, DBD, LBD | 168.855  5.411  5.734 | 166.048  6.937  7.015 | 170.601  4.593  4.806 |
| get_dihedral  comDBD, last_DBD_atom, first_LBD_atom, comLBD | -157.251 | -143.641 | -166.304 |
| ss%  DBD  LBD | Helix  : 30.67%  Sheet  :  9.33%  Loop  :  0.00%  Helix  : 67.29%  Sheet  :  4.21%  Loop  :  0.00% | Helix  : 34.67%  Sheet  :  9.33%  Loop  :  0.00%  Helix  : 69.55%  Sheet  :  4.09%  Loop  :  0.00% | Helix  : 40.00%  Sheet  :  9.33%  Loop  :  0.00%  Helix  : 70.80%  Sheet  :  3.98%  Loop  :  0.00% |

**Table 3. Comparison of human LXRB experimental (PDB: 4NQA) and predicted structures (AlphaFold2)**

|  | Experimental (LXRB-4NQA) | | Predicted (LXRB -AlphaFold2) |
| --- | --- | --- | --- |
|  | Chain B | Chain I |  |
| super exp, predicted  (all-atom, CA, backbone)  DBD  LBD | 0.538 (397 to 397)  0.433 (61 to 61)  0.423 (230 to 230)  0.520 (1519 to 1519)  0.479 (222 to 222)  0.474 (879 to 879) | 0.656 (439 to 439)  0.614 (68 to 68)  0.615 (266 to 266)  0.380 (1460 to 1460)  0.315 (207 to 207)  0.324 (823 to 823) |  |
| get_distance (comDBD-comLBD) | 45.331 Angstroms | 43.537 Angstroms | 43.928 Angstroms |
| get_angle (com) | 95.267 | 94.161 | 94.880 |
| get_dihedral  comDBD, last_DBD_atom, first_LBD_atom, comLBD | -26.436 | -39.143 | -39.260 |
| DBD ss%  LBD ss% | Helix  : 38.46%  Sheet  :  8.97%  Loop  :  0.00%  Helix  : 73.73%  Sheet  :  2.54%  Loop  :  0.00% | Helix  : 38.46%  Sheet  :  8.97%  Loop  :  0.00%  Helix  : 72.46%  Sheet  :  2.54%  Loop  :  0.00% | Helix  : 46.15%  Sheet  :  8.97%  Loop  :  0.00%  Helix  : 74.15%  Sheet  :  2.97%  Loop  :  0.00% |

Lower DBD RMSD for chain B and lower LBD RMSD for chain I. Predicted structure is more similar to chain I in terms of DBD-LBD distance, angles. Predicted structure has slightly higher helix content in DBD (46.15% vs 38.46%).

**Table 4. Comparison of human NURR1 vs experimental (PDB: 7wnh) and predicted structures (AlphaFold2)**

|  | Experimental (NURR1-7WNH | | | | Predicted (NURR1-AlphaFold2) |
| --- | --- | --- | --- | --- | --- |
|  | Chain A | Chain B | Chain C | Chain D |  |
| super exp, predicted  (all-atom, CA, backbone)  DBD  LBD | 0.411 (427 to 427)  0.333 (66 to 66)  0.363 (251 to 251)  0.819 (1325 to 1325)  0.713 (184 to 184)  0.725 (734 to 734) | 0.415 (429 to 429)  0.326 (65 to 65)  0.368 (251 to 251)  0.709 (1377 to 1377)  0.592 (191 to 191)  0.602 (764 to 764) | 0.409 (422 to 422)  0.319 (65 to 65)  0.348 (245 to 245)  1.014 (1431 to 1431)  0.908 (199 to 199)  0.904 (783 to 783) | 0.416 (439 to 439)  0.306 (65 to 65)  0.369 (259 to 259)  0.635 (1393 to 1393)  0.556 (201 to 201)  0.567 (802 to 802) |  |
| get_distance (comDBD-comLBD) | 37.72 Angstroms | 36.93 Angstroms | 63.191 Angstroms | 65.044 Angstroms | 44.956 Angstroms |
| get_angle (com)  comDBD, comHinge, comLBD | 115.893 | 112.096 | 134.403 | 135.500 | 120.367 |
| get_dihedral  comDBD, last_DBD_atom, first_LBD_atom, comLBD | 108.236 | 103.090 | -105.849 | -103.796 | -103.432 |
| ss%  DBD  LBD | Helix content: 41.10%  Sheet content: 0%  Loop content: 58.90%  Helix content: 69.12%  Sheet content: 4.15%  Loop content: 26.73% | Helix content: 41.10%  Sheet content: 0%  Loop content: 58.90%  Helix content: 69.78%  Sheet content: 4%  Loop content: 26.22% | Helix content: 41.10%  Sheet content: 0%  Loop content: 58.90%  Helix content: 71.56%  Sheet content: 4.13%  Loop content: 24.31% | Helix content: 36.99%  Sheet content: 0%  Loop content: 63.01%  Helix content: 70.35%  Sheet content: 3.98%  Loop content: 25.66% | Helix content: 36.99%  Sheet content: 0%  Loop content: 63.01%  Helix content: 70.87%  Sheet content: 3.91%  Loop content: 25.22% |

**Table 5. Comparison of human PPARG experimental (PDB: 3DZY, 3DZU, 3E00) and predicted structures**

|  | Experimental (PPARG-3DZY, 3DZU, 3E00) | | | Predicted (PPARG -AlphaFold2) |
| --- | --- | --- | --- | --- |
|  | 3DZY | 3DZU | 3E00 |  |
| super exp, predicted  (all-atom, CA, backbone)  DBD  LBD | 0.444 (451 to 451)  0.362 (69 to 69)  0.376 (262 to 262)  0.422 (1519 to 1519)  0.336 (217 to 217)  0.361 (864 to 864) | 0.544 (460 to 460)  0.452 (70 to 70)  0.438 (269 to 269)  0.501 (1543 to 1543)  0.419 (223 to 223)  0.440 (879 to 879) | 0.475 (451 to 451)  0.334 (67 to 67)  0.379 (260 to 260)  0.436 (1560 to 1560)  0.355 (224 to 224)  0.378 (880 to 880) |  |
| get_distance (comDBD-comLBD) | 39.162 Angstroms | 38.657 Angstroms | 39.368 Angstroms | 39.343 Angstroms |
| get_angle | 85.959 | 85.232 | 84.956 | 84.193 |
| get_dihedral  comDBD, last_DBD_atom, first_LBD_atom, comLBD | 56.500 | 55.722 | 55.295 | 57.849 |
| DBD ss%  LBD ss% | Helix: 37.33%  Sheet:  9.33%  Loop:  0.00%  Helix: 67.60%  Sheet:  4.80%  Loop:  0.00% | Helix: 30.67%  Sheet:  9.33%  Loop:  0.00%  Helix: 67.84%  Sheet:  4.71%  Loop:  0.00% | Helix: 29.33%  Sheet:  9.33%  Loop:  0.00%  Helix: 67.05%  Sheet:  4.65%  Loop:  0.00% | Helix: 36.00%  Sheet:  9.33%  Loop:  0.00%  Helix: 71.05%  Sheet:  4.51%  Loop:  0.00% |

When superimposed, 3DZY, 3E00 and predicted structure from AlphaFold has slightly lower RMSD values compared to 3DZU for both DBD and LBD.

No much difference for DBD-LBD distance and angles.

3DZY and predicted structure has similar helix content in their DBD, while it is lower for 3DZU and 3E00. LBD ss% is similar within experimental structures, but slightly higher in predicted structure. (Note: there are some missing amino acids in the middle of LBD of 3DZY (17), 3DZU (12), 3E00 (9)).

Mid* - com of last residue of DBD and first residue of LBD.

**Table 6. Comparison of human RARB experimental (PDB: 5UAN) and predicted structures (AlphaFold2)**

|  | Experimental (RARB-5UAN) | Predicted (RARB -AlphaFold2) |
| --- | --- | --- |
|  | Chain B |  |
| super exp, predicted  (all-atom, CA, backbone)  DBD  LBD | 0.318 (394 to 394)  0.234 (55 to 55)  0.262 (219 to 219)  0.304 (1452 to 1452)  0.257 (213 to 213)  0.263 (834 to 834) |  |
| get_distance (comDBD-comLBD) | 35.374 Angstroms | 35.143 Angstroms |
| get_angle (com) | 109.476 | 110.474 |
| get_dihedral  comDBD, last_DBD_atom, first_LBD_atom, comLBD | 117.318 | 114.942 |
| ss%  DBD  LBD | Helix  : 36.36%  Sheet  : 10.61%  Loop  :  0.00%  Helix  : 65.67%  Sheet  :  3.86%  Loop  :  0.00% | Helix  : 40.91%  Sheet  : 10.61%  Loop  :  0.00%  Helix  : 70.82%  Sheet  :  3.86%  Loop  :  0.00% |

Low CA and backbone RMSD (<0.3) for both DBD and LBD. Similar distance, angles. Predicted structure has slightly higher helix content in both DBD and LBD (40.91% vs 36.36%, 70.82% vs  65.67%).

**Table 7A. Comparison of human RXRA experimental (PDB: 3DZY, 3DZU, 3E00) and predicted structures (AlphaFold2)**

|  | Experimental (RXRA-3DZY, 3DZU, 3E00) | | | Predicted (RXRA -AlphaFold2) |
| --- | --- | --- | --- | --- |
|  | 3DZY | 3DZU | 3E00 |  |
| super exp, predicted  (all-atom, CA, backbone)  DBD  LBD | 0.348 (414 to 414)  0.291 (66 to 66)  0.298 (251 to 251)  0.484 (1265 to 1265)  0.406 (184 to 184)  0.434 (742 to 742) | 0.348 (413 to 413)   0.288 (62 to 62)  0.298 (239 to 239)  0.495 (1299 to 1299)  0.429 (191 to 191)  0.443 (745 to 745) | 0.366 (419 to 419)  0.294 (62 to 62)  0.291 (236 to 236)  0.447 (1307 to 1307)  0.354 (187 to 187)  0.367 (727 to 727) |  |
| get_distance (comDBD-comLBD) | 46.520 Angstroms | 47.458 Angstroms | 46.528 Angstroms | 48.139 Angstroms |
| get_angle | 71.757 | 73.897 | 71.947 | 121.176 |
| get_dihedral  comDBD, last_DBD_atom, first_LBD_atom, comLBD | 80.140 | 79.962 | 84.028 | 83.265 |
| ss%  DBD  LBD | Helix: 36.36%  Sheet: 10.61%  Loop: 0.00%  Helix: 66.67%  Sheet:  0.00%  Loop:  0.00% | Helix: 36.36%  Sheet: 10.61%  Loop:  0.00%  Helix: 73.08%  Sheet:  0.00%  Loop:  0.00% | Helix: 36.36%  Sheet:  0.00%  Loop:  0.00%  Helix: 70.19%  Sheet:  4.33%  Loop:  0.00% | Helix: 40.91%  Sheet: 10.61%  Loop:  0.00%  Helix: 74.24%  Sheet:  3.93%  Loop:  0.00% |

Superimposed (LBD) 3E00 and predicted structure has slightly lower RMSD values compared to 3DZY, 3DZU structures. DBD-LBD distance is similar between experimental and predicted structures, but the angle they form is different (get_angle). Predicted structure has slightly higher helix content in both DBD and LBD (40.91% vs 36.36%, 74.24% vs 66.67-73.08%). (Note: there are some missing amino acids in the middle of LBD of 3DZY (23), 3DZU (22), 3E00 (22)).

**Table 7B. Comparison of human RXRA experimental (PDB: RXRA-4NQA (A, H), 5UAN) and predicted structures**

|  | Experimental (RXRA-4NQA (A, H), 5UAN) | | | Predicted (RXRA -AlphaFold2) |
| --- | --- | --- | --- | --- |
|  | 4NQA (chain A) | 4NQA (chain H) | 5UAN |  |
| super exp, predicted  (all-atom, CA, backbone)  DBD  LBD | 0.503 (406 to 406)  0.399 (55 to 55)  0.454 (229 to 229  0.439 (1383 to 1383)  0.390 (198 to 198)  0.397 (790 to 790) | 0.429 (367 to 367)  0.297 (52 to 52)  0.329 (207 to 207)  0.419 (1310 to 1310)  0.370 (194 to 194)  0.372 (759 to 759) | 0.405 (327 to 327)  0.359 (49 to 49)  0.363 (194 to 194)  0.456 (1246 to 1246)  0.403 (184 to 184)  0.399 (734 to 734) |  |
| get_distance (comDBD-comLBD) | 57.113 Angstroms. | 58.627 Angstroms. | 54.117 Angstroms. | 48.139 Angstroms. |
| get_angle | 132.470 | 119.218 | 151.459 | 121.176 |
| get_dihedral  comDBD, last_DBD_atom, first_LBD_atom, comLBD | 148.117 | -121.145 | -113.138 | 83.265 |
| ss%  DBD  LBD | Helix : 40.91%  Sheet: 0.00%  Loop:  0.00%  Helix: 67.11%  Sheet:  0.00%  Loop:  0.00% | Helix: 34.85%  Sheet:  0.00%  Loop:  0.00%  Helix: 69.91%  Sheet:  4.17%  Loop:  0.00% | Helix: 43.64%  Sheet:  0.00%  Loop:  0.00%  Helix: 70.33%  Sheet:  3.35%  Loop:  0.00% | Helix: 40.91%  Sheet: 10.61%  Loop:  0.00%  Helix: 74.24%  Sheet:  3.93%  Loop:  0.00% |

Experimental and predicted structure have different DBD-LBD distances. Dihedral angle is different. Similar DBD helix content between 4NQA chain A, 5UAN and predicted structure. Sheet content?? ((Note: there are some missing amino acids in the middle of LBD of chain H of 4NQA (14) and 5UAN (21). 5UAN is also missing DBD residues in the middle (11).
