# Supplementary material for: Bridging prediction and reality: Comprehensive analysis of experimental and AlphaFold 2 full-length nuclear receptor structures
Source: Comput Struct Biotechnol J. 2025 May 15;27:1998–2013. doi: 10.1016/j.csbj.2025.05.010 (PMC12149446; doi:10.1016/j.csbj.2025.05.010)
Supplement: MMC 5 — Data for Molprobity and Ramachandran plot analysis. [file mmc5.pdf]

MolProbity Ramachandran analysis

7prv_B_GR.pdb, model 1

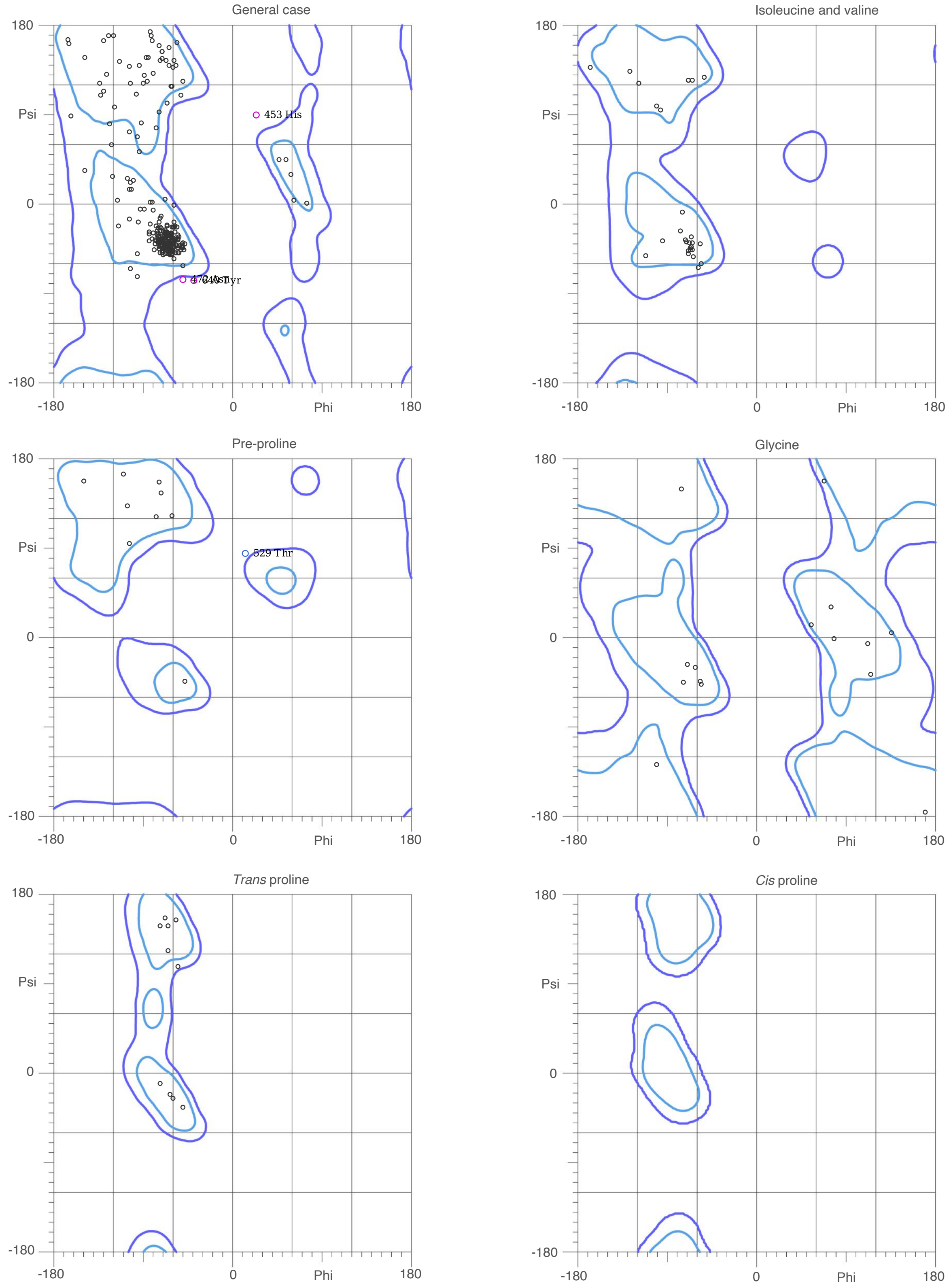

92.4% (293/317) of all residues were in favored (98%) regions.
98.7% (313/317) of all residues were in allowed (>99.8%) regions.

There were 4 outliers (phi, psi):
- 453 His (25.0, 90.1)
- 472 Asn (-50.6, -76.9)
- 529 Thr (13.6, 85.3)
- 640 Tyr (-39.3, -77.0)

Lovell, Davis, et al. Proteins 50:437 (2003)

MolProbity Ramachandran analysis

AF-P04150-F1_GR_converted_match_7prv_B.pdb, model 1

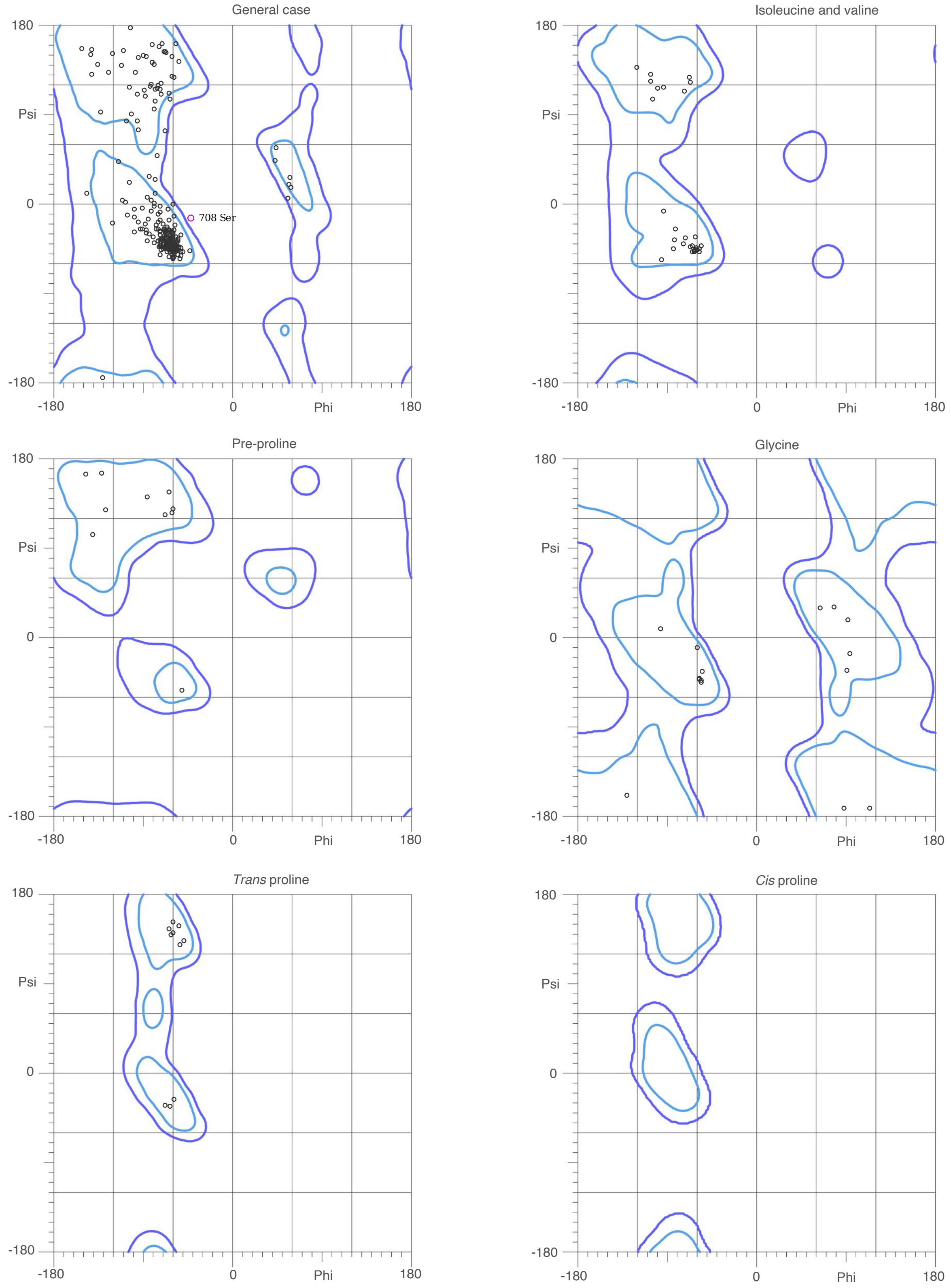

96.8% (307/317) of all residues were in favored (98%) regions.
99.7% (316/317) of all residues were in allowed (>99.8%) regions.

There were 1 outliers (phi, psi):
708 Ser (-42.6, -15.0)

MolProbity Ramachandran analysis

4iqr_B_HNF4A.pdb, model 1

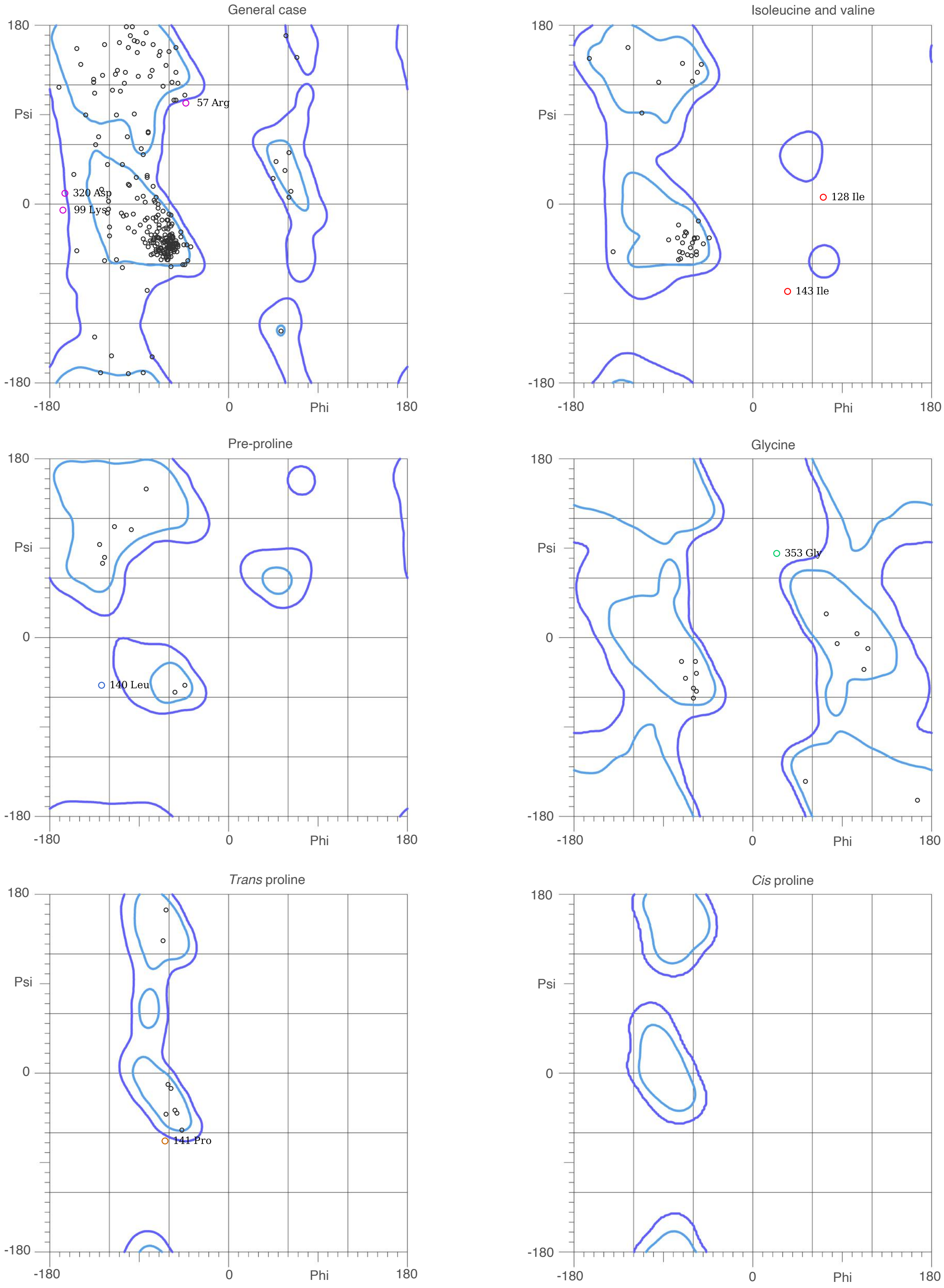

87.5% (266/304) of all residues were in favored (98%) regions.
97.4% (296/304) of all residues were in allowed (>99.8%) regions.

There were 8 outliers (phi, psi):
57 Arg (-43.6, 102.5)
99 Lys (-167.4, -6.7)
128 Ile (71.3, 7.3)
140 Leu (-128.6, -48.3)
141 Pro (-64.4, -68.8)
143 Ile (35.9, -88.9)
320 Asp (-165.5, 11.9)
353 Gly (24.6, 85.3)

Lovell, Davis, et al. Proteins 50:437 (2003)

MolProbity Ramachandran analysis

AF-P41235-F1_HNF4A_converted_match_4iqr_B1.pdb, model 1

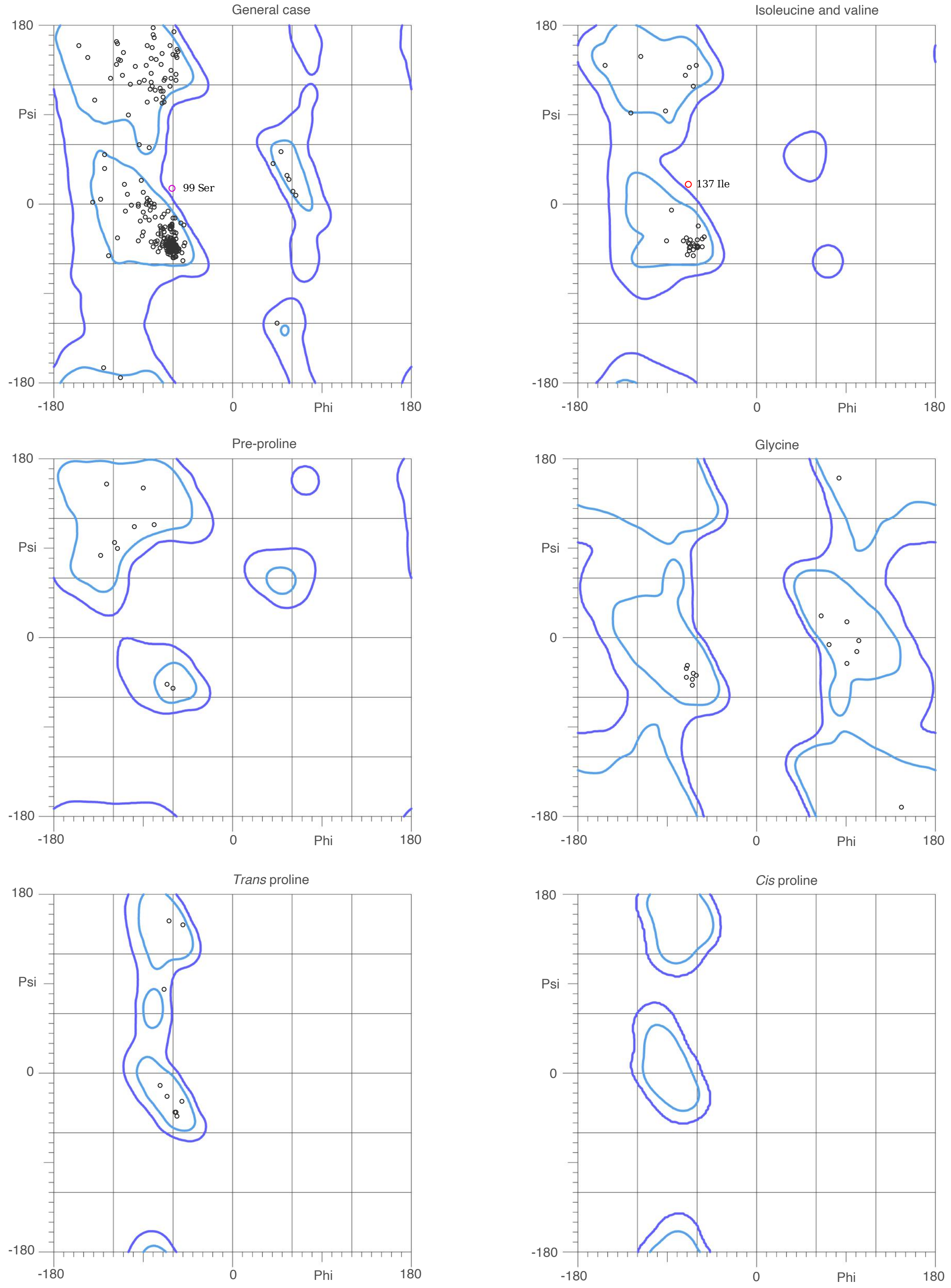

96.1% (292/304) of all residues were in favored (98%) regions.
99.3% (302/304) of all residues were in allowed (>99.8%) regions.

There were 2 outliers (phi, psi):
99 Ser (-61.3, 16.8)
137 Ile (-69.3, 20.3)

MolProbity Ramachandran analysis

4nqa_B_LXRB.pdb, model 1

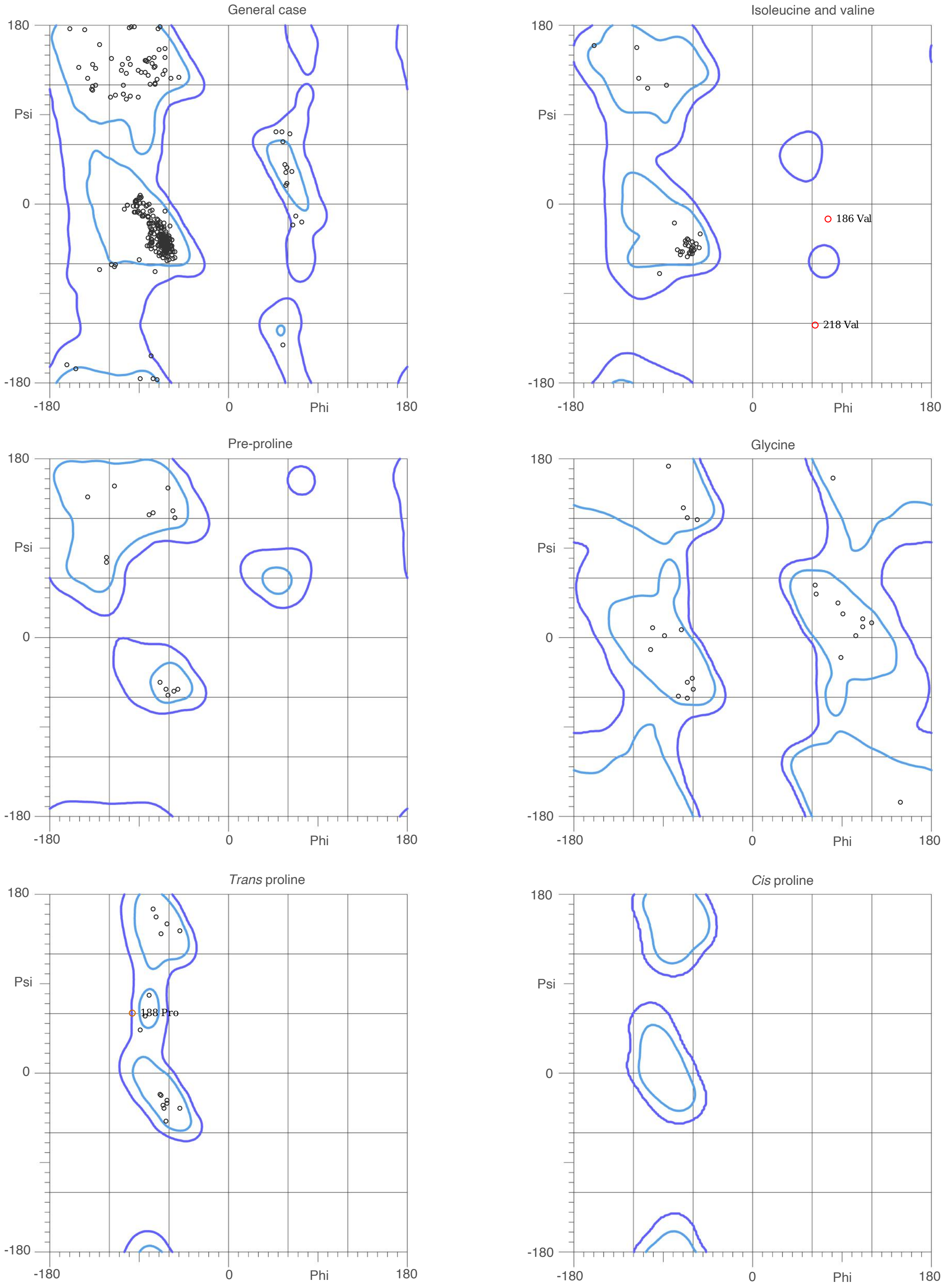

94.2% (344/365) of all residues were in favored (98%) regions.
99.2% (362/365) of all residues were in allowed (>99.8%) regions.

There were 3 outliers (phi, psi):
- 186 Val (76.2, -15.7)
- 188 Pro (-97.7, 61.8)
- 218 Val (63.9, -122.5)

MolProbity Ramachandran analysis

AF-P55055-F1_LXRB_converted_match_4NQA_B.pdb, model 1

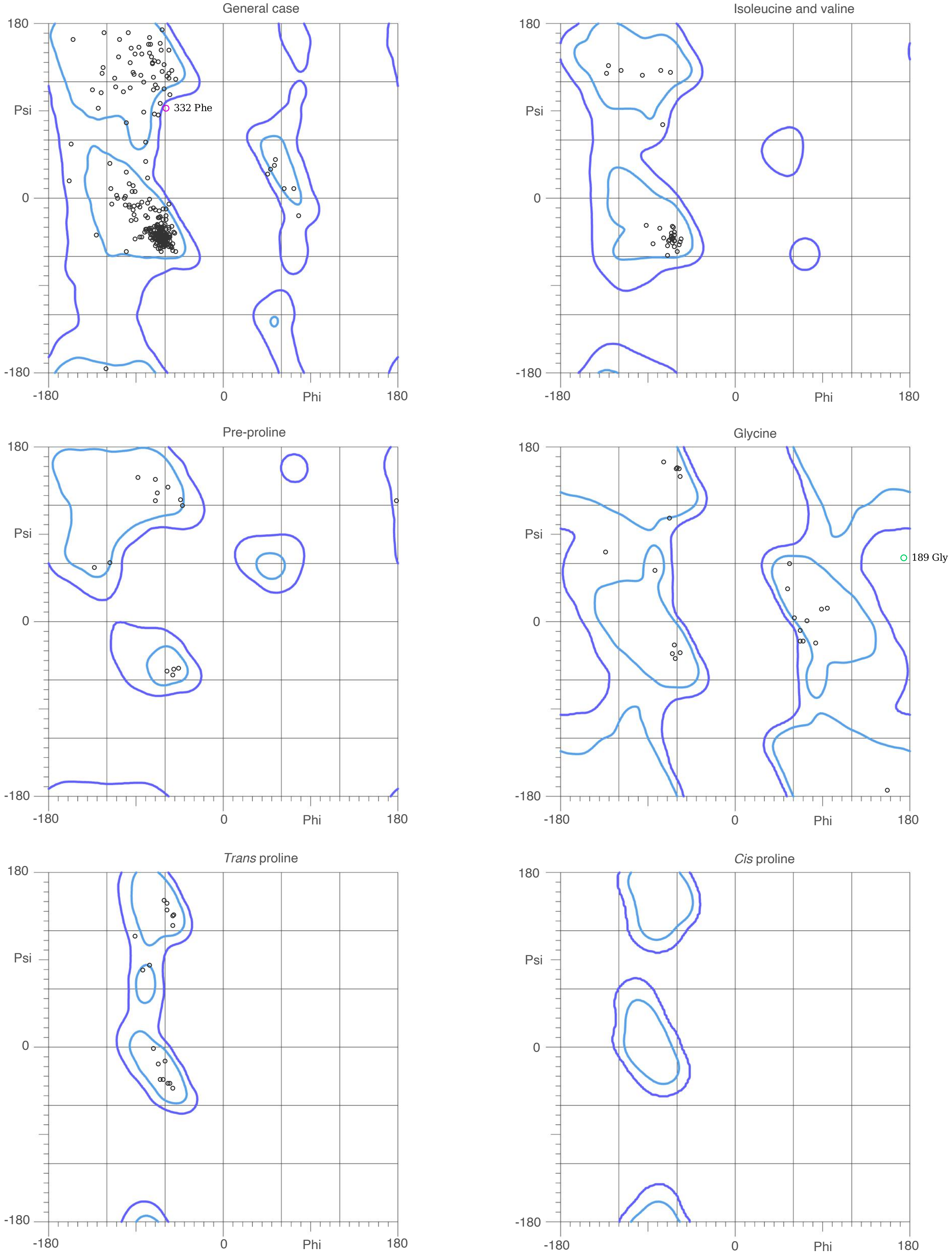

93.4% (340/364) of all residues were in favored (98%) regions.
99.5% (362/364) of all residues were in allowed (>99.8%) regions.

There were 2 outliers (phi, psi):
189 Gly (175.0, 66.7)
332 Phe (-59.7, 93.1)

Lovell, Davis, et al. Proteins 50:437 (2003)

MolProbity Ramachandran analysis

7wnh_B_NURR1.pdb, model 1

General case

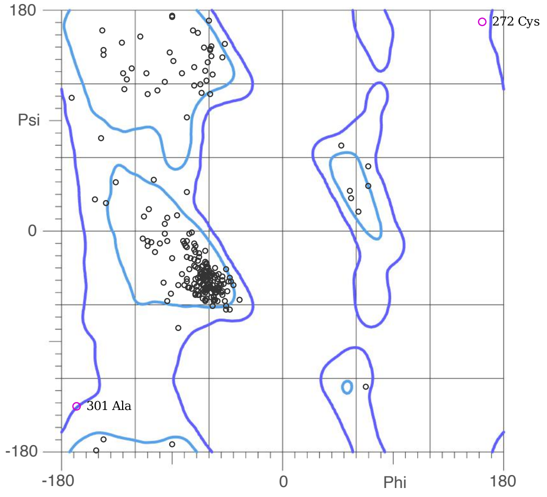

Isoleucine and valine

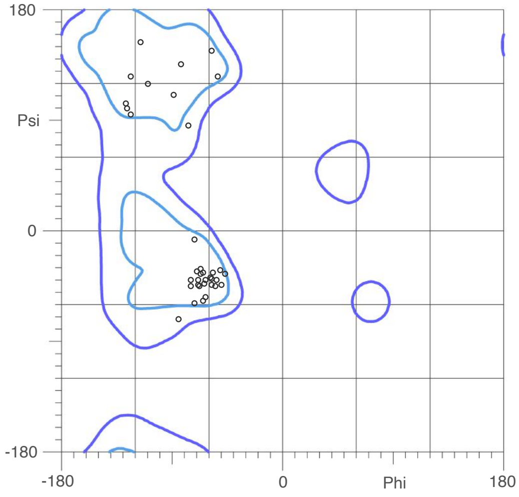

Pre-proline

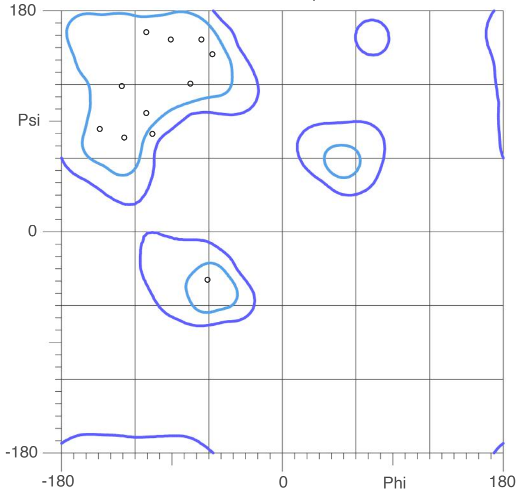

Glycine

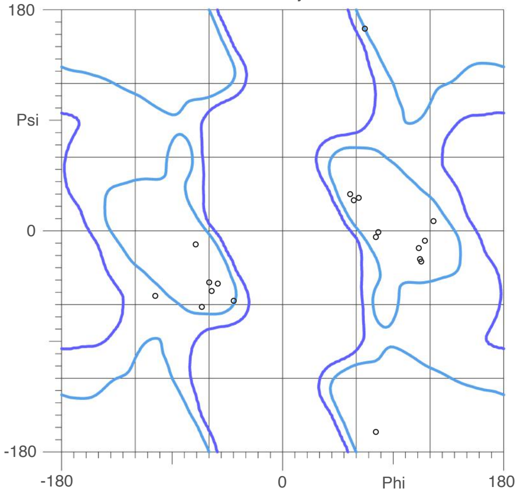

Trans proline

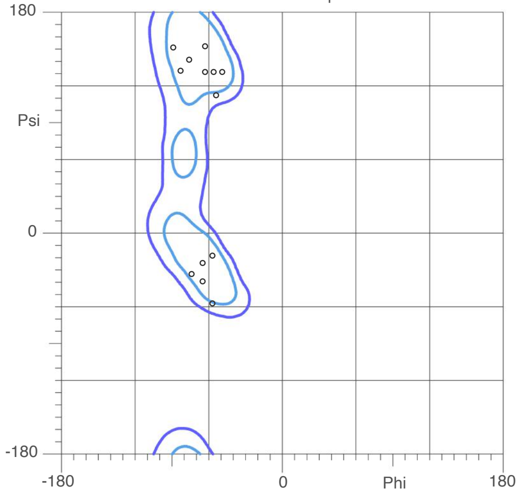

Cis proline

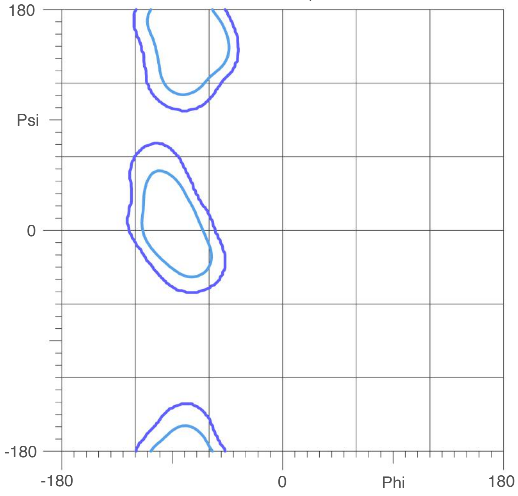

91.5% (281/307) of all residues were in favored (98%) regions.
99.3% (305/307) of all residues were in allowed (>99.8%) regions.

There were 2 outliers (phi, psi):
272 Cys (163.8, 171.8)
301 Ala (-169.0, -143.6)

MolProbity Ramachandran analysis

AF-P43354-F1_NURR1_converted_match_7wnh_B_1_.pdb, model 1

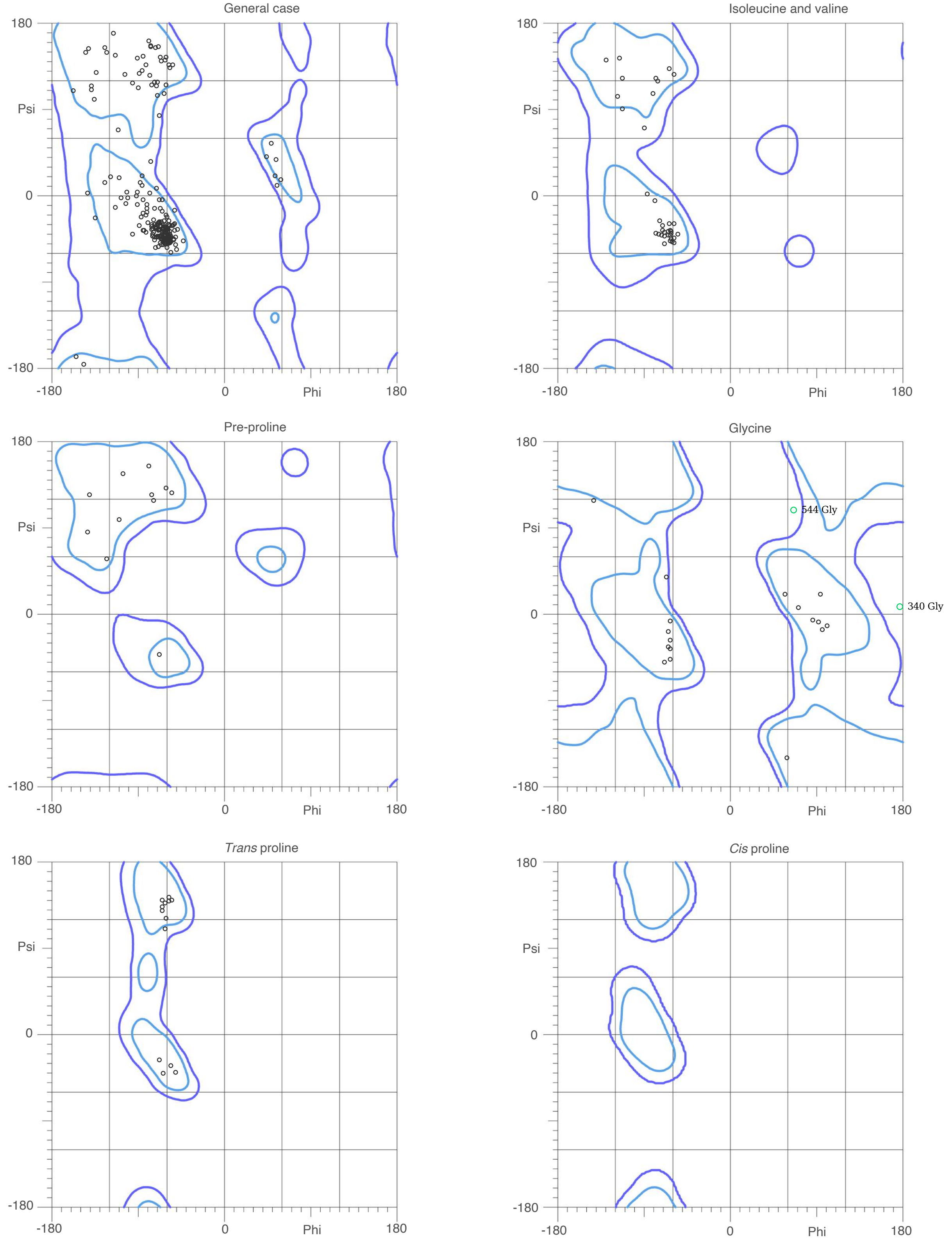

94.8% (291/307) of all residues were in favored (98%) regions.
99.3% (305/307) of all residues were in allowed (>99.8%) regions.

There were 2 outliers (phi, psi):
340 Gly (177.0, 8.5)
544 Gly (66.3, 109.5)

MolProbity Ramachandran analysis

3e00_D_PPARG.pdb, model 1

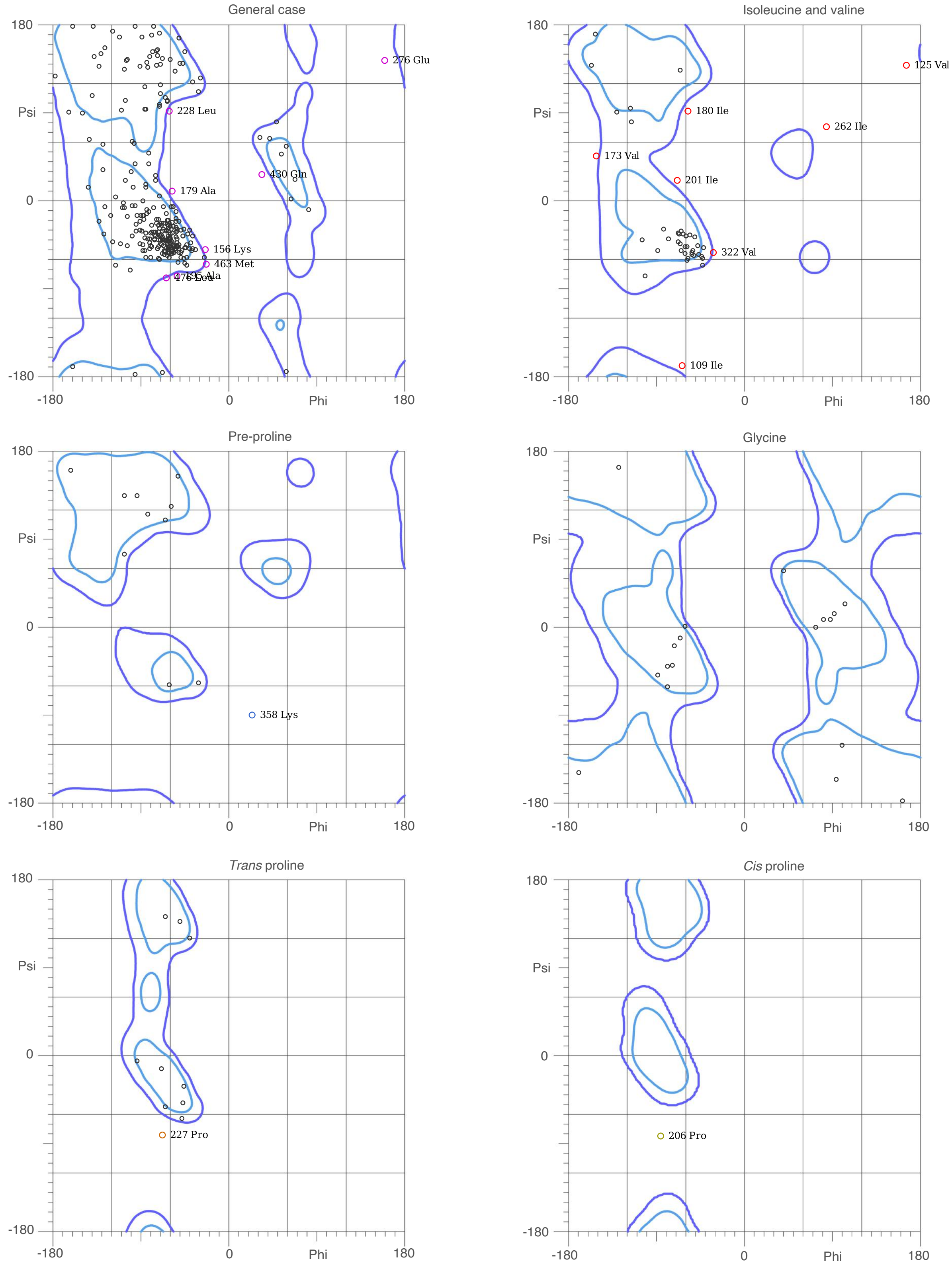

78.0% (280/359) of all residues were in favored (98%) regions.
95.0% (341/359) of all residues were in allowed (>99.8%) regions.

There were 18 outliers (phi, psi):

109 Ile (-64.3, -169.8)	206 Pro (-86.8, -82.3)	463 Met (-23.2, -65.2)
125 Val (166.9, 139.8)	227 Pro (-68.4, -81.2)	476 Leu (-64.9, -79.1)
156 Lys (-24.3, -50.7)	228 Leu (-61.5, 92.6)	
173 Val (-152.5, 46.7)	262 Ile (84.1, 76.6)	
179 Ala (-58.2, 10.2)	276 Glu (160.7, 144.5)	
180 Ile (-59.0, 92.7)	322 Val (-32.8, -53.2)	
195 Ala (-53.5, -77.6)	358 Lys (24.5, -90.8)	
201 Ile (-69.7, 21.7)	430 Gln (34.9, 27.4)	

http://kinemage.biochem.duke.edu

Lovell, Davis, et al. Proteins 50:437 (2003)

MolProbity Ramachandran analysis

AF-P37231-F1_PPARG_converted_match_3e00.pdb, model 1

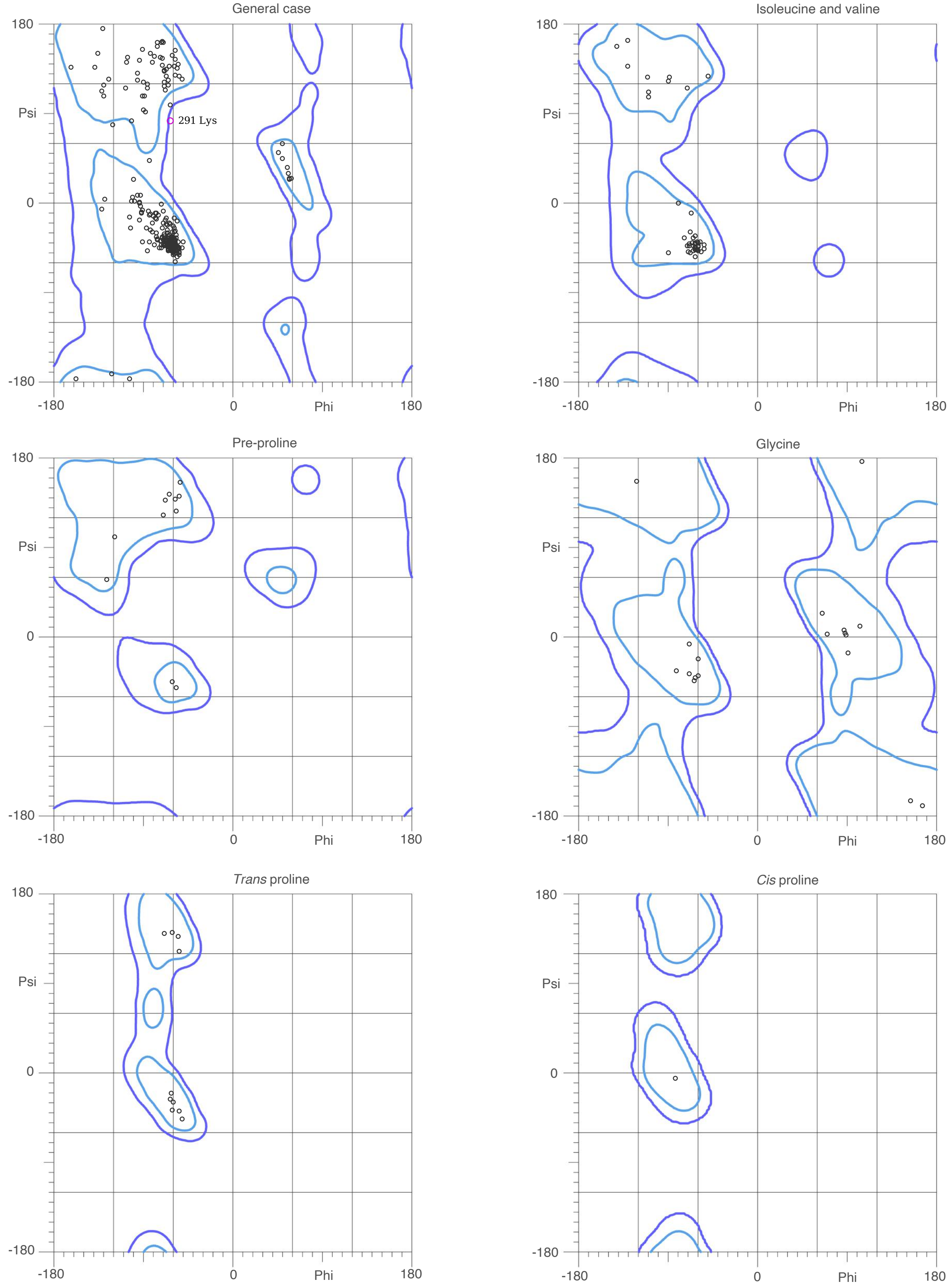

98.9% (355/359) of all residues were in favored (98%) regions.
99.7% (358/359) of all residues were in allowed (>99.8%) regions.

There were 1 outliers (phi, psi):
291 Lys (-63.1, 83.0)

MolProbity Ramachandran analysis

5uan_B_RARB.pdb, model 1

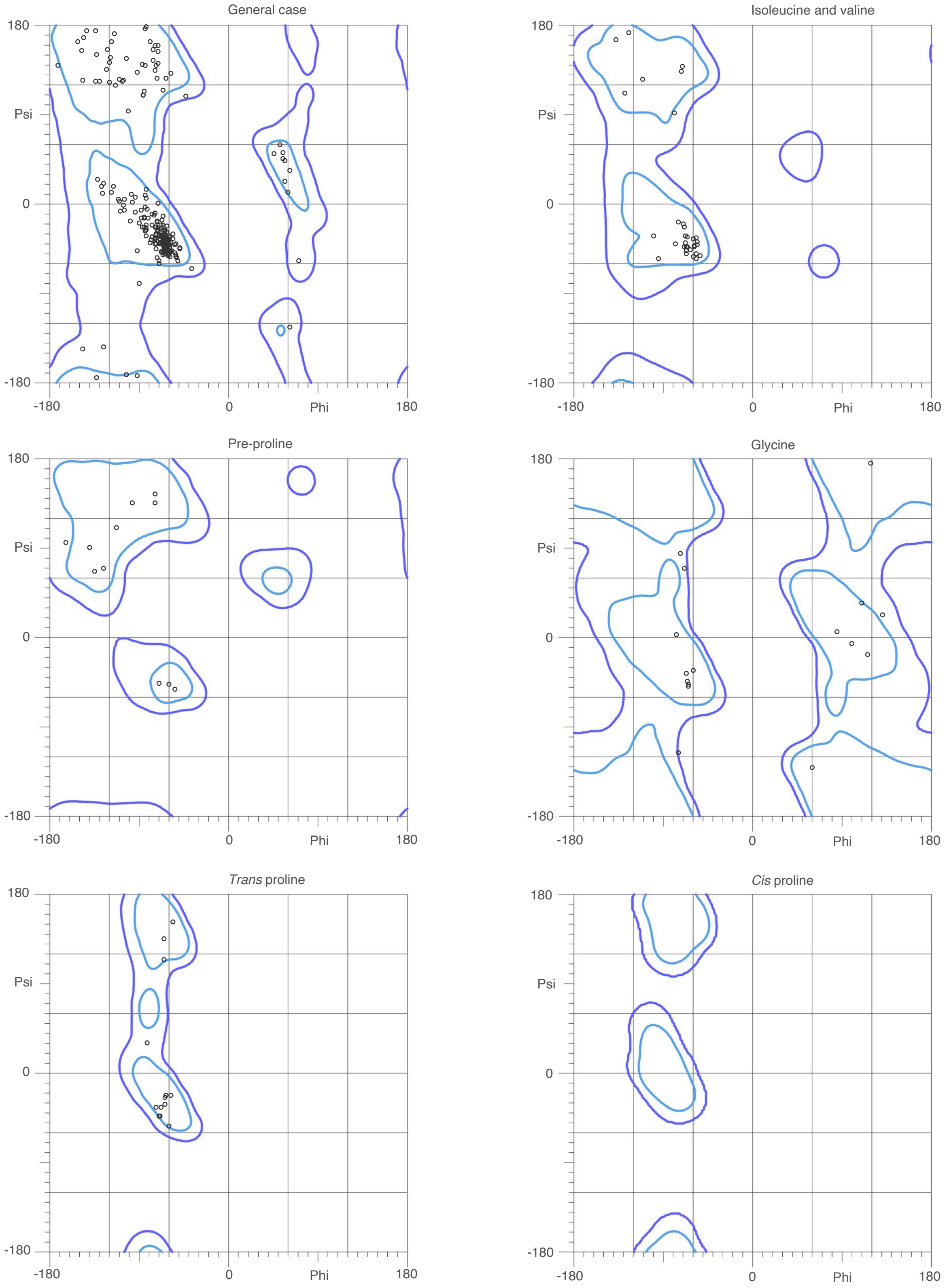

94.7% (288/304) of all residues were in favored (98%) regions.
100.0% (304/304) of all residues were in allowed (>99.8%) regions.

There were no outliers.

MolProbity Ramachandran analysis

AF-P10826-F1_RARB_converted_match_5uan_B.pdb, model 1

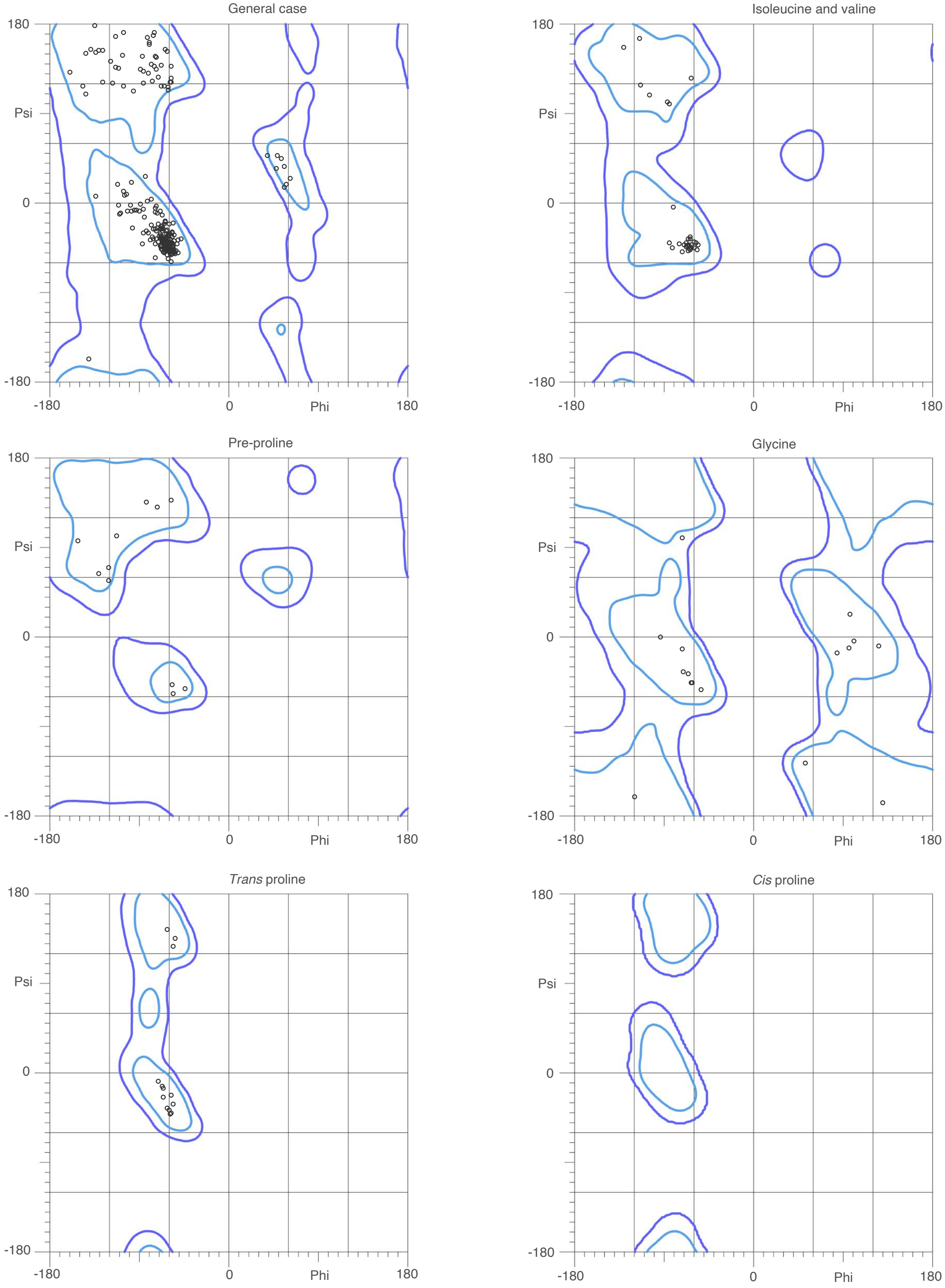

98.7% (300/304) of all residues were in favored (98%) regions.
100.0% (304/304) of all residues were in allowed (>99.8%) regions.

There were no outliers.

MolProbity Ramachandran analysis

3dzy_A_RXRA.pdb, model 1

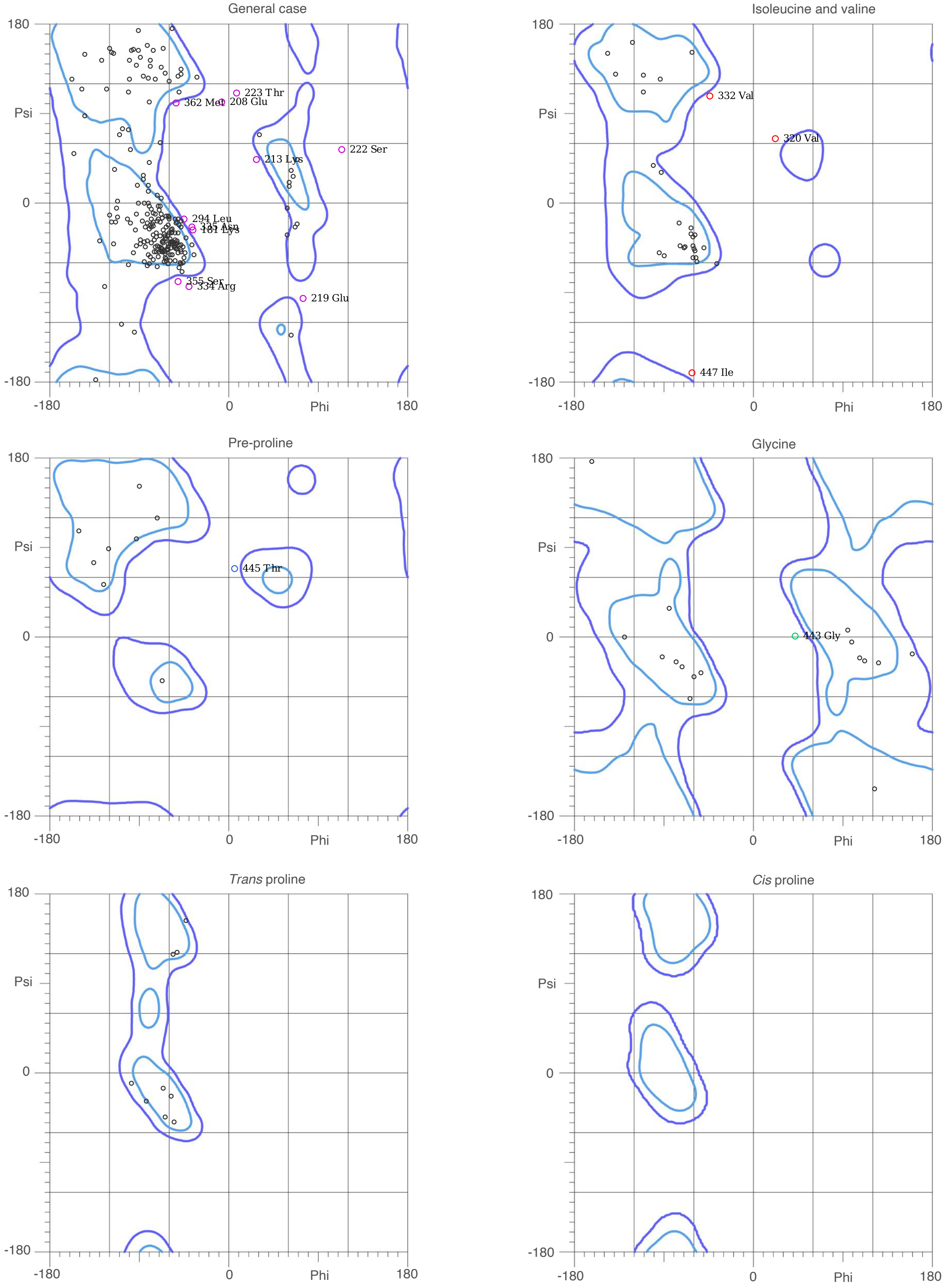

78.5% (234/298) of all residues were in favored (98%) regions.
94.6% (282/298) of all residues were in allowed (>99.8%) regions.

There were 16 outliers (phi, psi):

181 Lys (-36.3, -27.3)	332 Val (-44.7, 108.6)
208 Glu (-7.5, 102.7)	334 Arg (-40.1, -84.3)
213 Lys (28.3, 44.2)	335 Asn (-37.1, -24.8)
219 Glu (76.0, -96.1)	355 Ser (-51.0, -79.5)
222 Ser (114.9, 54.2)	362 Met (-53.9, 101.6)
223 Thr (8.3, 111.3)	443 Gly (42.0, 1.4)
294 Leu (-46.0, -16.4)	445 Thr (6.8, 69.0)
320 Val (22.5, 65.1)	447 Ile (-62.3, -171.8)

MolProbity Ramachandran analysis

AF-P19793-F1_RXRA_converted_match_3dzy_A.pdb, model 1

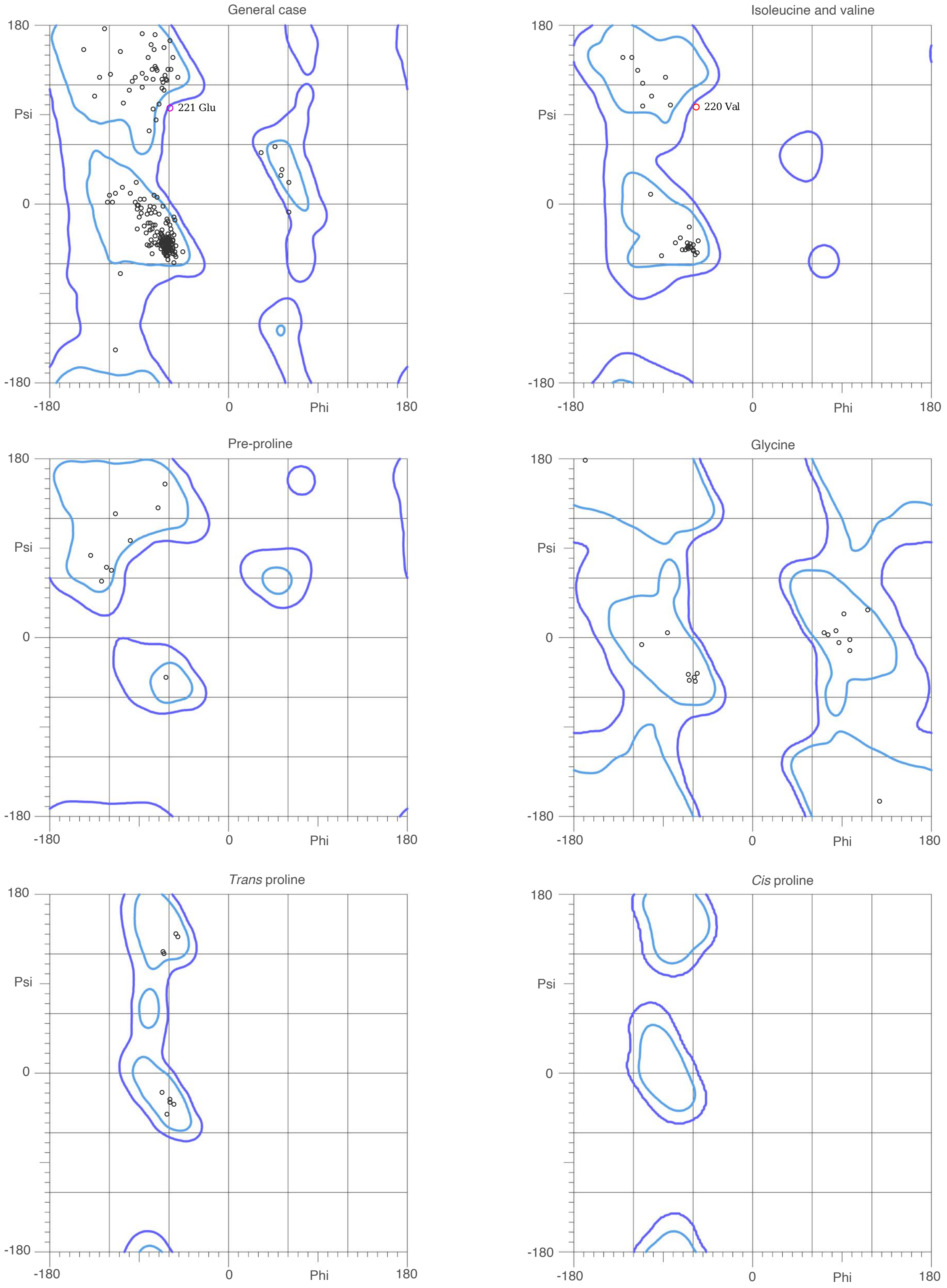

96.6% (288/298) of all residues were in favored (98%) regions.
99.3% (296/298) of all residues were in allowed (>99.8%) regions.

There were 2 outliers (phi, psi):
220 Val (-57.3, 98.4)
221 Glu (-59.2, 97.6)

Lovell, Davis, et al. Proteins 50:437 (2003)
